# Development and validation of an immune and stromal prognostic signature in uveal melanoma to guide clinical therapy

**DOI:** 10.18632/aging.103779

**Published:** 2020-10-26

**Authors:** Qianwen Gong, Qi Wan, Anqi Li, Yubin Yu, Xiangyu Ding, Lei Lin, Xiaoliang Qi, Liang Hu

**Affiliations:** 1 School of Ophthalmology and Optometry, Eye Hospital, Wenzhou Medical University, Wenzhou, Zhejiang, China; 2 State Key Laboratory of Ophthalmology, Zhongshan Ophthalmic Center, Sun Yat-Sen University, Guangzhou, China

**Keywords:** uveal melanoma, immune, stromal, prognosis

## Abstract

The tumor microenvironment is known to play an important role in uveal melanoma. Reliable prognostic signatures are needed to aid high risk patients and improve prognosis. Uveal melanoma tissues from three public datasets were analyzed. RNA sequence data of uveal melanoma and corresponding clinical features were obtained from The Cancer Genome Atlas database. Immune and stromal scores were calculated by applying the “ESTIMATE” algorithm. The samples were divided into high and low immune or stromal score groups. We constructed prognostic models by using the ‘lasso’ package and tested them for 500 iterations. The cell signature was validated in another GSE44295 and GSE84976 datasets. We found that the median survival time of the low immune/stromal score group is longer than that of the high-score group. Thirteen immune cells and one stromal cell were concerned significant in predicting poor overall survival rate. Finally, a four-cell model was identified. Further validation revealed that the low-risk group has a significantly better survival than the high-risk group in another two datasets (*P* < 0.05). Moreover, the high-risk group is more sensitive to immunotherapy and chemotherapy. Summarizing, the proposed immune cells signature is a promising biomarker for estimating overall survival in uveal melanoma.

## INTRODUCTION

Uveal melanoma (UM) is the most common type of malignant tumor of the adult eye, and 50% of patients with UM will eventually die as a result [1–3]. The prognosis for patients with UM remains poor, though there have been some certain advances in the diagnosis and treatment of UM [4]. Thus, there is an urgent need in this advancing field to further enhance prognostic accuracy and provide an efficient therapy [5].

In recent years, with the rapid development of immunotherapy, it has been reported that the tumor microenvironment (TME) plays a pivotal role in cancer progression and therapeutic responses [6–7]. Prognostic or predictive biomarkers related to TME may hold great promise in identifying molecular targets and guiding patient management [8].

In the context of the tumor microenvironment, immune and stromal cells are two major types suggested as crucial for the diagnostic and prognostic assessment of tumors [9]. An increasing body of literature suggests that immune cell infiltration may co-evolve with the sequential genetic changes occurring in UM [10–13]. Early changes resulting in a gain of chromosome 8q are reported to activate macrophage infiltration, while sequential loss of BRCA1-associated protein-1 (BAP1) expression could drive T cell infiltration in UM [12]. Although increasing numbers of studies have explored the microenvironment using differentially expressed genes, a comprehensive analysis with an overall landscape is still lacking. Fortunately, the availability of public large-scale datasets, such as the cancer genome atlas (TCGA), could be used to gain numerous amounts of RNA sequencing (RNA-seq) data to represent the tumor microenvironment [14–15]. And Yoshihara et al. designed the “ESTIMATE” (Estimation of STromal and Immune cells in MAlignant Tumor tissue using Expression data) algorithm [16]. By analyzing specific gene expression signature of immune and stromal cells, immune and stromal scores can be calculated using the ESTIMATE algorithm to predict the infiltration of non-tumor cells. In recent years, the ESTIMATE algorithm has been reported to be applied in breast cancer, glioblastoma multiforme, etc., proving the effectiveness of such big-data-based algorithms [9, 17, 18]. Effective use of all this information would be helpful in improving clinical management.

In this study, we used a number of datasets with the ESTIMATE algorithm to identify the influence of immune and stromal cells in UM patients, and to develop and validate a prognostic signature to better guide the therapy and prognosis of UM.

## RESULTS

### Subgroup analysis of immune scores and stromal scores

**Figure 1:**
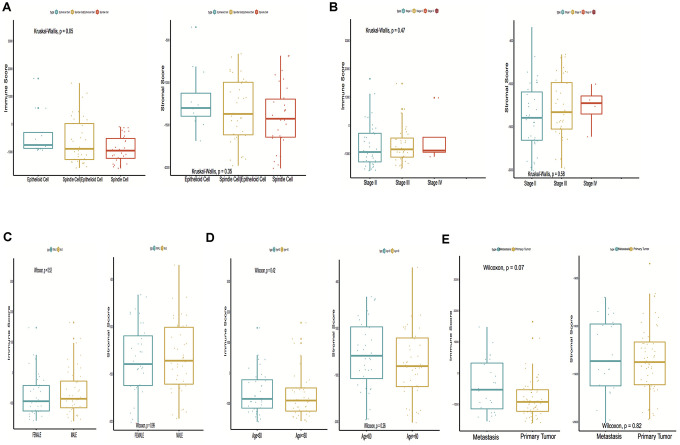
**Immune scores and stromal scores are associated with Uveal Melanoma (UM) clinical subtypes, like histological type, metastasis, etc.** (**A**) Distribution of immune scores and stromal scores of histological subtypes, Box-plot shows that there is a significant association between UM subtypes and the level of immune scores (*P* = 0.05); (**B**) Distribution of immune scores and stromal scores of stage subtypes; (**C**) Distribution of immune scores and stromal scores of sex subtypes; (**D**) Distribution of immune scores and stromal scores of age subtypes; (**E**) Distribution of immune scores and stromal scores of metastasis subtypes. Box-plot shows that there is no significant association between other subtypes and the levels of immune and stromal scores.

**Figure 2:**
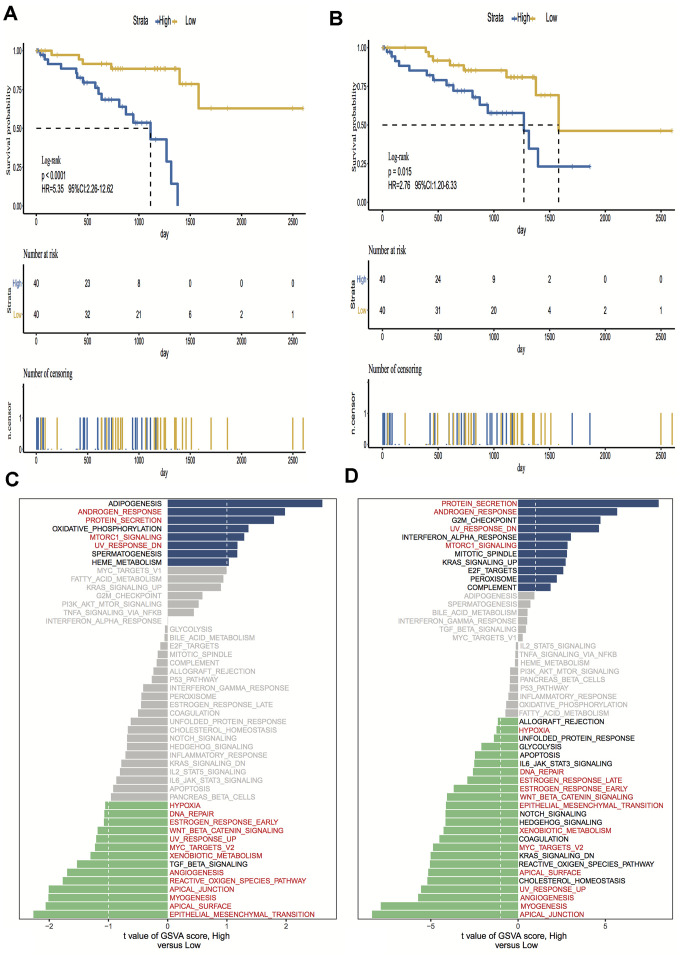
**Kaplan-Meier survival analysis and Gene set variation analysis (GSVA) of high vs. low immune scores/stromal scores groups.** (**A**, **B**) Overall survival among patients with uveal melanoma (UM) based on their immune and stromal scores; (**C**, **D**) Differential pathway activities between high and low immune and stromal scores groups, the same pathways are marked in red in the immune and stromal groups. Hazard ratios (HRs) and 95% CIs are for high vs low immune and stromal risk. The log-rank test was used to calculate P values in comparing risk groups.

A total of 15,187 generally changed mRNA expression values and clinicopathological characteristics of UM were obtained from TCGA. Based on the ESTIMATE algorithm, immune scores were distributed between -1600 and 1645 and stromal scores ranged from -2011 to -348, respectively. A subgroup analysis of clinical characteristics showed that only histological type has a significant difference in immune scores (Figure 1A). The subtype cases of epithelioid cells had the highest immune scores (*P* = 0.05). The clinical characteristics of stage, gender and age are statistically insignificant (Figures 1B–1D). Moreover, the metastatic UM showed a higher immune and stromal score, but this was statistically insignificant compared with the primary melanoma (Figure 1E). To determine the potential correlation of overall survival with immune scores and stromal scores, we divided the 80 UM samples of high and low immune or stromal score groups by median value. Kaplan-Meier survival curves show that the overall survival of samples with low immune scores and stromal scores is longer than that of the samples in the high score group (hazard ratio [HR], 5.35 [*P* < 0.001]; and 2.76 [*P* = 0.02], respectively) (Figure 2A, 2B).

### Gene set variation analysis

To investigate the hallmark pathways shared by different immune or stromal groups, we performed gene set variation analysis (GSVA). According to the following criteria of *P* value < 0.05 and |GSVA score| ≥ 1, four hallmark terms were commonly differently expressed in the high immune and stromal score group, and 12 hallmark terms were commonly differently regulated in the low immune and stromal score group. The same pathways are marked in red in Figure 2C, 2D.

### Clustering for immune and stromal cells infiltration

**Figure 3:**
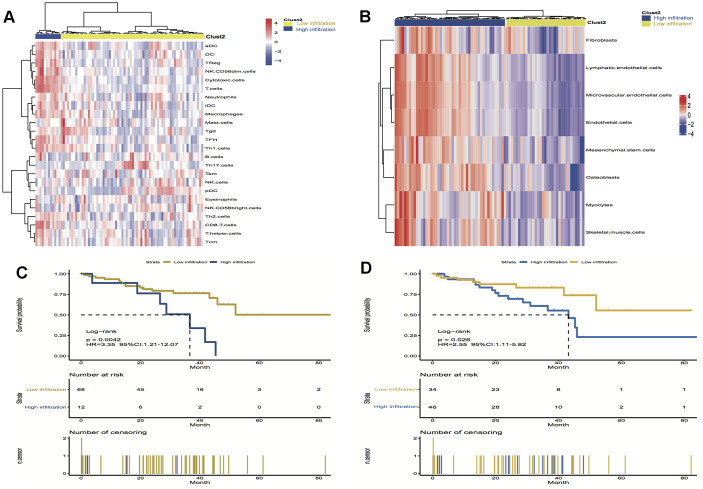
**Unsupervised clustering of immune and stromal cells for 80 patients in the UM cohort.** (**A**, **B**) The low infiltration and high infiltration cells in immune and stromal group; (**C**) Kaplan-Meier curves for overall survival (OS) of UM patients show that immune infiltration patterns are significantly associated with overall survival (log-rank test, *P* = 0.004); (**D**) Kaplan-Meier curves for OS of UM patients show that stromal infiltration patterns are significantly associated with overall survival (log-rank test, *P* = 0.03). Hazard ratios (HRs) and 95% CIs are for high or low immune and stromal infiltration risk.

To validate the above findings, the immune and stromal cells phenotypes expression profiles from UM were optimally clustered by applying the “ClassDiscovery” algorithm and the results are shown in Figures 3A, 3B. Cell infiltration in immune and stromal subtypes showed that the overall survival of the high infiltration group is significantly shorter than the low infiltration group (HR, 3.35; *P* = 0.004; and HR, 2.55; *P* = 0.03, respectively; Figure 3C, 3D).

### Identification and confirmation of prognostic model

**Figure 4:**
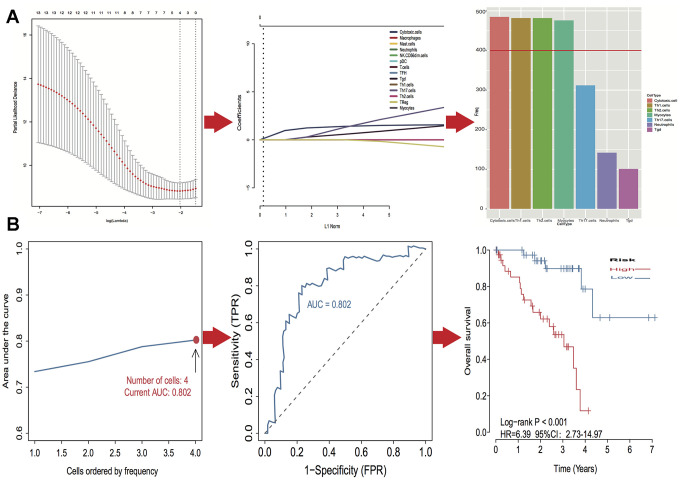
**Identification of optimal cell signature for overall survival (OS) prediction.** (**A**) The process of building the signature containing four cell types (3 immune cells and 1 stromal cell) and the coefficients calculated using the lasso method: from 500 iterations of lasso-penalized multivariate modeling, four cell types were reported as optimal for survival prediction more than 400 times; (**B**) The AUC curves of cell type models and Kaplan–Meier survival analysis of four-cell-type model in TCGA. Then the number of cells is four, the value of AUC is the highest (0.802). Kaplan–Meier curves indicated that there is a significant difference between high- and low-risk groups (log-rank *P* < 0.001).

**Figure 5:**
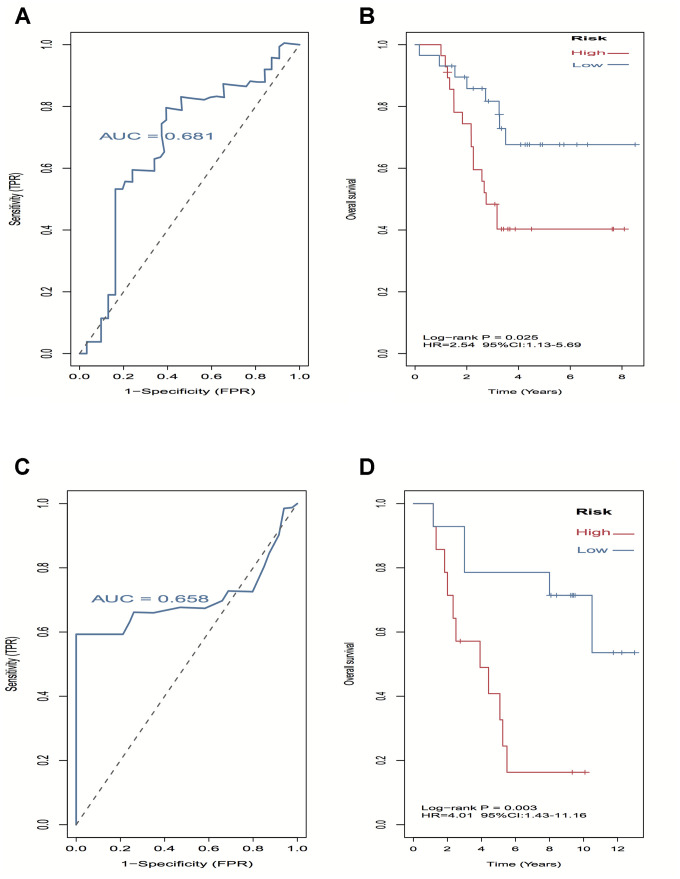
**External validation of four-cell-type model in GSE44295 and GSE84976 datasets.** (**A**–**C**) The AUC curves in GSE44295 and GSE84976 datasets. (**B**, **D**) Kaplan-Meier survival analysis in GSE44295 and GSE84976 datasets, revealed that the low-risk groups have a significantly better survival than the patients in high-risk group.

**Figure 6:**
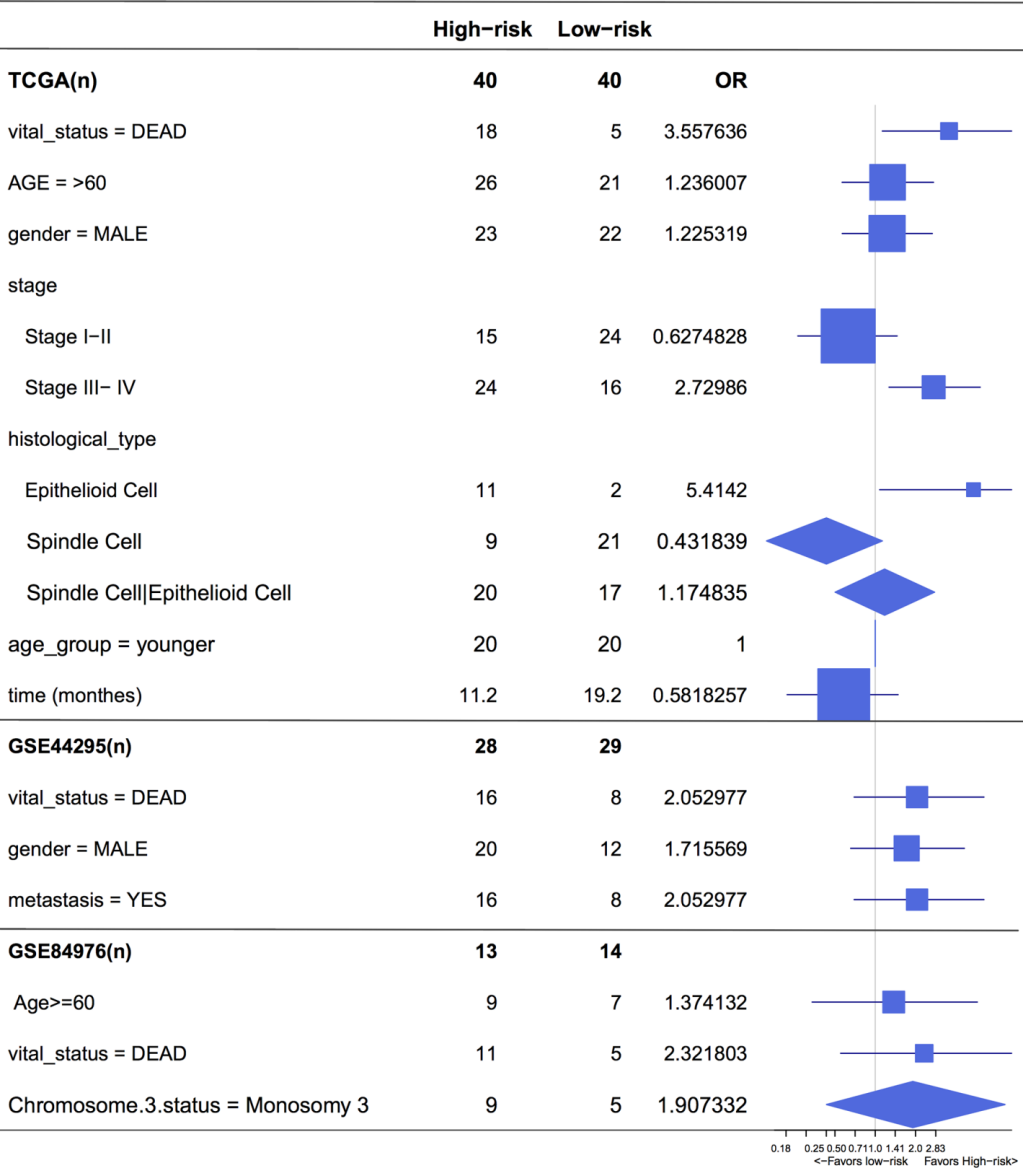
**Forest plot for the odds ratio (OR) of high or low 4-cell-type model related risk groups.** The length of the horizontal line corresponds to the confidence interval, and the size of the OR data marker is inversely proportional to the confidence interval. The vertical dotted line indicates OR of 1.0.

A log-rank test of Kaplan-Meier survival curves of 24 immune cells and 11 stromal cells showed that there was a total of 13 immune cells and 1 stromal cell capable of significantly predicting poor overall survival rate (*P* < 0.05). Moreover, we used LASSO modeling with 500 iterations to evaluate associations between the 14 selected cells and overall survival in the TCGA dataset. Finally, a four-cell (cytotoxic cells, Th1 cells, Th2 cells and myocytes cells) biomarker was screened out of the 14 selected cells to build a risk signature based on the criteria (Figure 4A). The risk score formula for overall survival was calculated as follows: risk score = 1.54 × (expression value of Cytotoxic cells) + 1.20 × (expression value of Th1 cells) + 2.80 × (expression value of Th2 cells) - 0.46× (expression value of Myocytes). The risk system calculates a risk score for each patient. Applying the median cut-off value of the risk scores, 80 patients with UM were divided into high-risk and low-risk groups. Kaplan-Meier curve indicated that there was a significant difference between high-risk and low-risk group (HR, 6.39; 95% confidence interval (CI), 2.73 to 14.97; *P* < 0.001) (Figure 4B). The area under the curve (AUC) values for the four-cell model was 0.802. To verify the predictive ability of the four-cell model, validation analysis was performed using the GSE44295 and GSE84976 datasets. The AUC values of four cells were 0.681 and 0.658, respectively (Figure 5A, 5C). The Kaplan-Meier curve revealed that the low-risk group have a significantly better survival than the patients in high-risk group with log-rank test (HR, 2.54; *P* = 0.03 and HR, 4.01; *P* = 0.003, respectively) (Figure 5B, 5D). Results of the subgroup analysis of clinical characteristics of low- and high- risk groups are shown in Figure 6.

### High-risk subgroup more sensitivite to immunotherapy and chemotherapy

**Figure 7:**
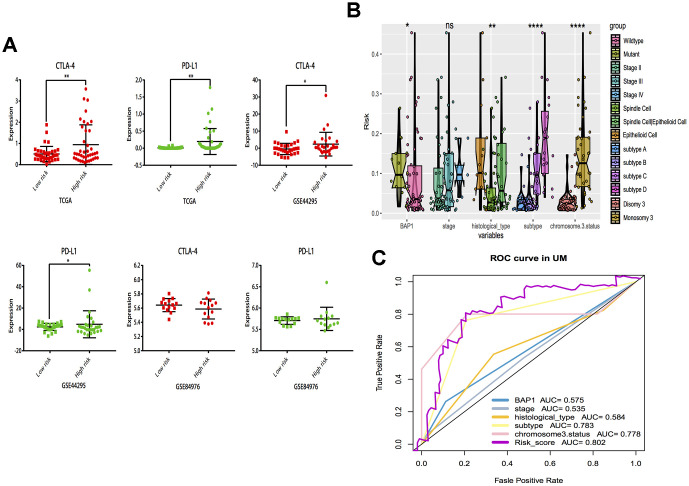
**Subgroup analysis and risk score distribution.** (**A**) Box-plot analysis of high and low groups in CTLA-4 and PD-L1 expression. (**B**) The association between risk score distribution and established prognostic markers, including BAP1 mutant, tumor stage, histological type, chromosome 3 status, and molecular subtype. (**C**) The 3 years area under the curve (AUC) of risk score and prognostic markers (BAP1 mutant, tumor stage, histological type, chromosome 3 status and molecular subtype) associated with OS in TCGA. *P < 0.05, ** *P* < 0.01,***P < 0.001,****P < 0.0001.

**Figure 8:**
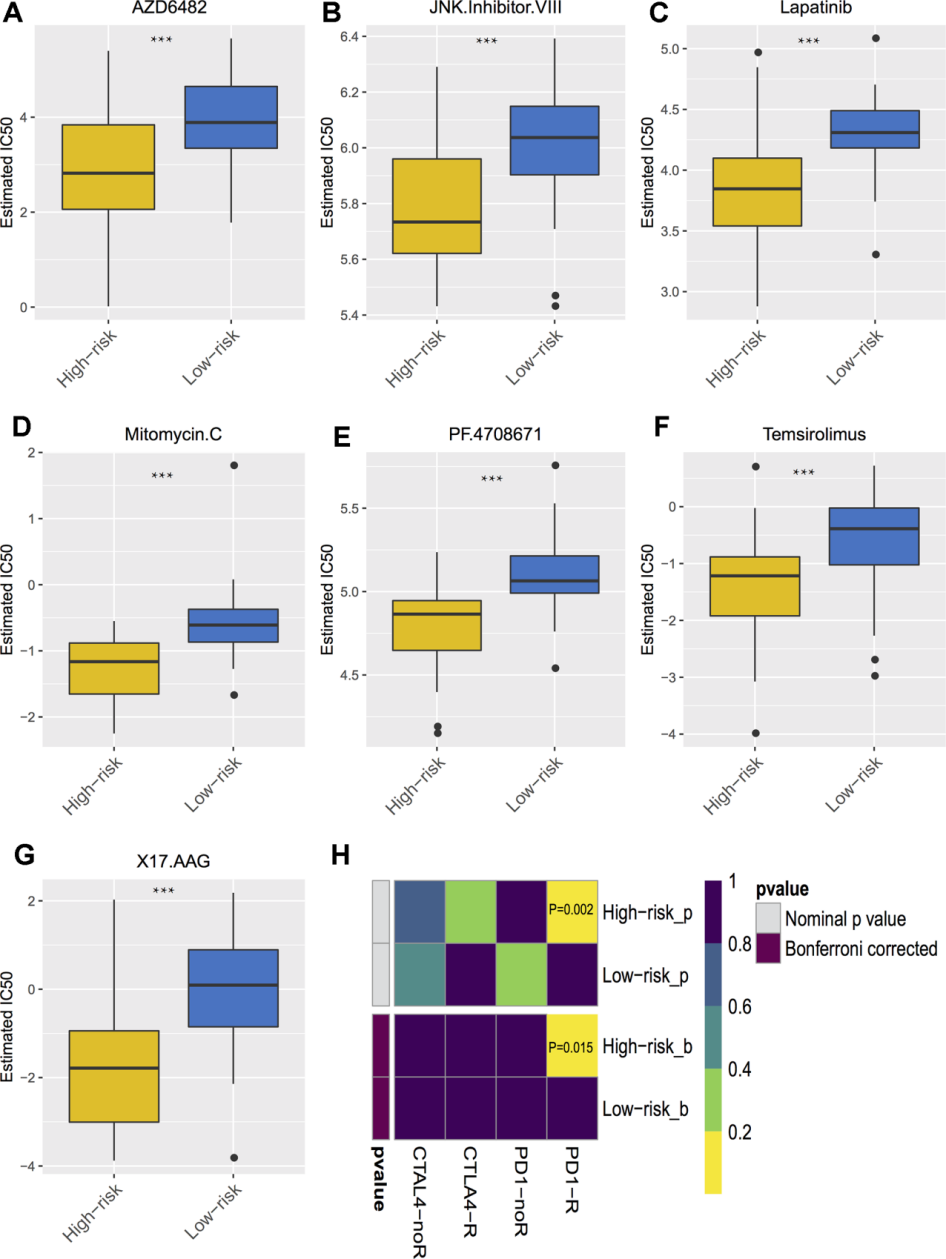
**Differential putative chemotherapeutic and immunotherapeutic response.** (**A**–**G**) The box plots of the estimated IC50 for the most sensitive chemotherapeutic drugs. (**H**) Submap analysis manifested that high risk group could be more sensitive to the programmed cell death protein 1 inhibitor (Bonferroni-corrected P = 0.02). *** *P* < 0.001.

The correlations of cytotoxic T-lymphocyte-associated protein 4 (CTLA-4) and programmed death-ligand 1 (PD-L1) expression with low- and high- risk groups were analyzed. The results revealed that the expression in the high-risk group was generally higher than that in the low-risk group (Figure 7A). The relationships between risk score and previously established prognostic markers, such as tumor stage, chromosome 3 status, mutated BAP1 and molecular subtype, were explored. The box plots in Figure 7B show that BAP1 mutant, subtype D, and monosomy 3 have a higher risk score than BAP1 wildtype, subtype A and disomy 3, respectively. Compared with the 3 years AUC values of these established prognostic markers (BAP1 mutant, tumor stage, histological type, subtype and chromosome 3 status), our signature can achieve higher accuracy value (Figure 7C). Furthermore, we used subclass mapping to compare the expression profile of the two subgroups and another dataset containing details of 47 patients with cutaneous melanoma that responded to immunotherapies, published in the TIDE website. Interestingly, we found that the high-risk group is more promising to respond to anti-PD-1 therapy (Bonferroni corrected *P* = 0.02) (Figure 8H). To further explore the response to chemotherapy between high- and low-risk patients with UM, we performed “pRRophetic” algorithm to estimate the chemotherapeutic response based on half maximal inhibitory concentration (IC50) available in the Genomics of Drug Sensitivity in Cancer (GDSC) database. Seven chemotherapeutic drugs, including AZD6482, JNK Inhibitor VIII, Lapatinib, Mitomycin C, PF.4708671, Temsirolimus and X17.AAG were identified as producing significant differences in the estimated IC50 between the high- and low-risk groups. Remarkably, we observed from the estimated IC50 of these chemotherapeutic drugs, that the high-risk group could be more sensitive to chemotherapies than those in low-risk group. (Figure 8A–8G).

## DISCUSSION

To the best of our knowledge, this is the first study to investigate the UM tumor microenvironment using the ESTIMATE algorithm based on large public datasets, developing and validating the contribution of one stromal and three immune cells signatures in UM prognosis. Our prognostic signature can further stratify clinically defined groups of patients (eg, age, stages I, II, III and IV UM, histological type) into subgroups with different risk analysis (Figure 6).

Previous studies mainly focused on the intrinsic genes of tumors [19], and some have provided elegant analyses on expression of immune-related genes or immune-infiltration in UM [11, 20]. However, a comprehensive analysis of the UM microenvironment consisting of larger cohorts is needed in studies of the UM microenvironment. In our study, we conduct the research with the help of numerous computational tools and public data, such as TCGA. The ESTIMATE algorithm was chosen for its compatible with RNA-Seq data and microarray data. The overall survival is correlated with immune scores and stromal scores, where the prognosis of low immune scores and stromal scores is better than that of the high-score group. TME infiltration patterns also show that the low immune and stromal infiltration pattern has a better prognosis than the high infiltration pattern, which is consistent with the immune and stromal scores.

On further investigation, we found 13 immune cells and 1 stromal cell capable of significantly predicting poor overall survival rate in log-rank test, but which cell is more responsible than another and which common pathway is involved? Finally, signatures for three immune cell types - cytotoxic cells, Th1 cells, and Th2 cells - together with one stromal cell type - myocytes - were identified. Similarly, previous studies have reported that CD4+ T lymphocytes was present in UM inflammatory infiltrates. Moreover, CD4+CD25+FoxP3+ Treg cells are capable of suppressing Th1 or Cytotoxic T lymphocytes responses and represent a major mechanism of tumor escape in several cancers [2]. Our pathway research also found that myogenesis is significantly expressed in both the low immune and low stromal groups, which is consistent with the cells identification. In cardiac myocytes research, it has been demonstrated that fibroblast growth factor-2 (FGF-2) synthesis can be regulated at the transcriptional level [21], and FGF-2 was found to rescue UM cells from growth inhibition by bromodomain and extraterminal protein inhibitors [22]. It has been suggested that co-targeting of FGF receptor signaling is required to increase the responses of metastatic UM to BET inhibitors [22], which is a point we could consider with the stromal findings. The epithelial-to-mesenchymal transition (EMT) investigated is also a common pathway in low immune and stromal group of UM, which is said to enable trans-differentiation of epithelial tumor cells, endowing them with migratory and invasive properties [23]. The EMT is demonstrated to be related to myogenesis [24]. Taken together, the myocyte expression is also an unusual phenomenon that deserves more attention and the risk score of prognostic signature is a tool that we could validate further in clinic.

All the three datasets showed that the correlation of CTLA-4 and PD-L1 expression with the low-risk group is significantly less than that of the high-risk groups. Thus, we were interested in investigating the response to treatment. It has been reported that the primary targets of immune checkpoint blockade (ICB) treatment are PD-1 and CTLA4 [25]. Previous studies have clarified that the success of anti-PD-1 and anti-CTLA-4 agents in UM has been much more limited [26–27]. Some of our results are consistent with the previous studies, but an interesting point is that the high-risk group is more responsive to anti-PD-1 therapy and several chemotherapeutic drugs. Furthermore, we checked several more drugs like mitomycin C and lapatinib, which could also be used as supplementary or combined treatment agents [28]. However, more benchwork and clinical studies are needed to further validation. As aforementioned, we first developed the prognostic model and calculated the risk score. It would be more helpful to identify patients at high-risk in clinic and attempt the sensitive immunotherapy and chemo-treatment.

In summary, our study reveals a comprehensive landscape of the immune and stromal microenvironment in UM, and provides a promising prognostic signature for UM. Patients with the high risk scores could benefit more from anti-PD-1 therapy and chemotherapy. Further investigations are needed to verify the accuracy in estimating prognoses and to test its clinical utility in patient management.

## MATERIALS AND METHODS

### RNA and clinical data

The RNA sequencing dataset and corresponding clinical follow-up information of UM were obtained from the TCGA database. This dataset was derived from the tissue samples from 80 adult patients, and an integrative analysis by UM area consortia has also been conducting used this dataset [29]. Survival time was regarded as the time from tissue removal to death. Another two UM datasets (GSE44295 and GSE84976), consisting of 85 UM samples from the Gene Expression Omnibus, were used as external validation sets.

### Tumor microenvironment estimation

Immune scores and stromal scores were calculated by applying the ESTIMATE algorithm in R package (“ESTIMATE”). By running ESTIMATE on TCGA RNA-Seq data, the immune scores and stromal scores of each uveal melanoma sample can be estimated. To quantify the proportions of immune and stromal cells in the UM samples, we first identified the biomarker genes of immune and stromal cells from the previously published articles [30, 31], and used single sample gene set enrichment analysis (ssGSEA) method to specifically discriminate 24 human immune cells and 11 stromal cells phenotypes using the R package (“GSVA”) to validate the ESTIMATE algorithm. The immune cells considered were dendritic cells (DCs), immature DCs (iDC), activated DCs (aDC), plasmacytoid DCs (pDC), natural killer (NK) cells, CD56dim NK cells, CD56bright NK cells, macrophages, neutrophils, eosinophils, mast cells, T cells, T central memory cells (Tcm), T effector memory cells, Tgd cells, CD8 T cells, regulatory T cells (Treg), T follicular helper cells (TFH), T helper cells, Th1, Th2, Th17, B cells, and cytotoxic cells. The stromal cells included fibroblasts, lymphatic endothelial cells, microvascular endothelial cells, endothelial cells, mesenchymal stem cells, osteoblasts, myocytes, and skeletal muscle cells.

### Gene set variation analysis

The uveal melanoma samples were divided into high vs. low immune score/stromal score groups by the median value. Then, GSVA was used to evaluate the common pathways shared in the tumor-infiltrating immune and stromal groups. These 50 hallmark pathways described in the molecular signature database, exported using the “GSEABase” package. Next, pathway activity estimates were assigned to individual samples by using the R package (“GSVA”).

### Heatmaps and clustering analysis

Heatmaps and clustering were generated using an R package (“pheatmap”).

### Survival analysis of immune and stromal cells

The best separation survival analysis of immune and stromal cells was performed using the “survminer” package. Kaplan-Meier were plotted and the differences among groups were compared using log-rank tests.

### Identification of prognostic model

The survival-related cells in primary selection are not suitable for clinical diagnosis. Therefore, a robust survival modeling approach was used to identify suitable cell signature. We constructed prognostic models by using the “lasso” package and ran the analysis for 500 iterations. Statistical stability under each model was evaluated and a frequency greater than 400 regarded as indicating a stable model. Kaplan–Meier survival curves were plotted and differences between the subgroups were compared using log-rank tests. Receiver operating characteristic curves [1] were drawn for the predicted 3-year overall survival (OS), and the AUC values was used to evaluate the specificity and sensitivity of the cell signature. Moreover, to prove the reliability of the result, this cell signature was further validated in another two independent datasets (GSE44295 and GSE84976).

### Immuno- and chemotherapeutic response prediction

To explore the likelihood of an immune- or chemotherapeutic response, we predicted the chemotherapeutic response for each sample based on the Genomics of Drug Sensitivity in Cancer (GDSC) database (https://www.cancerrxgene.org) [32]. The most significant chemotherapeutic drugs were selected (*P* < 0.0001). The prediction process was implemented using the R package “pRRophetic”. Although immune checkpoint inhibitors have not yet been approved as routine drugs for UM, we also predicted the likelihood of response to immunotherapy by using the TIDE website tool (http://tide.dfci.harvard.edu/) [25].

### Statistical analysis

All statistical analyses were conducted using the R package (version 3.5.2). For comparisons of two groups, the statistical significance for normally distributed variables was estimated using Student’s t tests, and non-normally distributed variables were analyzed using Mann-Whitney U tests (also called the Wilcoxon rank-sum test). For comparisons of more than two groups, Kruskal-Wallis tests and one-way analysis of variance were used as non-parametric and parametric methods, respectively. The association between cell signature and clinicopathological characteristics was analyzed using Fisher’s exact test.
